# What is the impact of fitness on injury risk during police academy training? A retrospective cohort study

**DOI:** 10.1186/s13102-020-00188-7

**Published:** 2020-07-06

**Authors:** Colin Tomes, Ben Schram, Rodney Pope, Robin Orr

**Affiliations:** 1 Faculty of Health Sciences and Medicine, Bond University, Robina, Australia; 2 Tactical Research Unit, Bond University, Robina, Australia; 3 Faculty of Science, Charles Sturt University, Bathurst, Australia

**Keywords:** Tactical, Attrition, Strength, Endurance, Injury risk, Law enforcement

## Abstract

**Background:**

In the conduct of their daily duties, law enforcement officers (LEO) are often required to perform dynamic, physically demanding tasks with little or no notice, sometimes at maximal levels of exertion. Given these requirements, training for prospective LEOs must be rigorous enough to ensure that when trainees graduate, they are competent in their response to crisis and resilient enough to maintain this for the span of their career. Therefore, based on previously reported effectiveness of fitness testing in predicting injury risk in predominantly military settings, the aim of this study was to investigate relationships between a physical ability test (PAT) and risk of injury during police recruit training.

**Methods:**

Retrospective PAT results and trainee injury records were obtained from a national police department and Mann-Whitney U tests were performed to investigate fitness differences between trainees who were, or were not, injured. Significant results were tested for effect size using Cliff’s delta (CD).

**Results:**

Significant differences in mean performance between groups existed for the following PAT components: pushups (injured mean 32.94 ± 8.66 reps, uninjured mean 35.67 ± 9.04 reps, *p* = 0.01 CD + 0.11) and right-hand grip strength (injured mean 49.61 ± 12.51 kg, uninjured mean 52.12 ± 11.17 kg, *p* = 0.042 CD + 0.22) for all injuries; vertical jump height (injured mean 51.75 ± 7.54 cm, uninjured mean 55.06 ± 8.19 cm, *p* = 0.032 CD + 0.41) for lower limb injuries, and all measures of grip strength for trunk injury.

**Conclusions:**

The results of this study suggest that a significant relationship between some PAT fitness components and injury risk exists during police recruit training.

## Background

In the conduct of their daily duties, law enforcement officers (LEO) are often required to perform dynamic, physically demanding tasks with little or no notice, sometimes at maximal levels of exertion [1]. In order to perform these occupational tasks safely and effectively, LEOs must not only be sufficiently fit but also resilient enough to perform these tasks regularly without experiencing excessive stress across a career [2]. Given these requirements, training for prospective LEOs must be rigorous enough to ensure that when trainees graduate, they are competent in their response to crisis and robust enough to maintain their capacity throughout the span of their career [2]. Such training includes not only mastery of the essential technical procedures required to safely and effectively promote public safety and enforce the law, but must also prepare prospective LEOs to face adversity, remain calm in dangerous emergency situations and make decisions when their safety and the safety of their communities is on the line.

Globally, as populations continue to age, the 18–24 year-old demographic Law Enforcement and other public defense agencies typically recruit from is shrinking, despite general increases in overall population [3]. Additionally, western obesity and inactivity epidemics are further limiting the pool from which applicants can be drawn. For example, in the United States, 31% of individuals 17–24 years old interested in enlistment are ineligible for military service due to obesity alone [3] and overall, 71% of this age group are ineligible for service for one or more health or fitness related reasons [3].

On joining a law enforcement agency (LEA), new trainees may be subject to environmental stressors (such as relocation and sharing close quarters with strangers), psychological stressors (such as academic pressure, and disrupted sleep), and physical stressors (such as a sudden increase in physical training, and a lack of recovery time). As such, trainees are at risk of physical overtraining and consequent injury and illness, or both [4]. These factors, while usually a mixture of deliberate and incidental impacts generally represent a substantial increase in mental and physical demand for most trainees [5]. For trainees with lower levels of fitness, the increased physical work requirement has an even greater impact, as these trainees must consistently work at a higher intensity relative to their own maximum to complete the same task when compared against more physically fit peers [6]. It is therefore not surprising that less fit trainees may be at a greater risk of injury than their fitter counterparts [7], who are themselves three to five times more likely to sustain an injury than their fully trained, operational counterparts [8].

Injuries in tactical training present a twofold problem for tactical organizations. Firstly, there are the intrinsic financial and time loss burdens the organization accrues [9]. Apart from the costs of any rehabilitation or compensation, it can cost an organization more than $85,000 AUD to identify a new trainee to replace one lost due to training injury [10]. This need to replace the trainee introduces a second problem; finding a suitable trainee from the aforementioned shrinking pool of potential applicants [3]. Hence it is in the best interests of LEA to identify, recruit and train candidates with the highest chance of successfully completing training.

Previous research, primarily on military trainees, has identified that a fixed-distance, timed run is effective in predicting musculoskeletal injury in a variety of settings [11, 12]. Other tests, mostly of muscle endurance, such as timed pushup [13, 14] and situp [12, 15] events, are less conclusive across studies but may still be valid predictors of injury in a police training setting. Two muscle fitness tests of strength (grip strength) and power (vertical jump) [6, 16] have been identified as predictors of not only injury, but other tactically relevant outcomes such as escalation of force incidents in operational LEO [17].

However, the relevance of the above research, associating performance on a physical fitness test with risk of injury during training, may be highly dependent upon the environment. For example, if one training academy completes a high volume of pushups as part of their training, pushup performance may be a greater predictor of injury risk than a 5 km run. The inverse may be true if the academy has a low pushup but high running requirement in its daily training. The disciplinary culture of an organization (assigning running laps vs. pushups or situps as punishment) may contribute as well. Therefore, based on the crucial need for LEAs to retain personnel recruited for training, and the previously reported utility of fitness testing for predicting risk of injury in a given environment, the aim of this study was to investigate relationships between components of a physical ability test (PAT) and risk of injury during police recruit training in a cohort of New Zealand (NZ) Police trainees.

## Methods

A cohort study was designed which analyzed data previously collected prospectively, from the NZ Police Constabulary Recruitment database. Trainee data were made non-identifiable before analysis and included age, height, weight, BMI, testing date, graduation result, injury status during training, and PAT score. Ethical approval for the study was provided by the Bond University Human Research Ethics Committee (BUHREC, Research Protocol BS02086).

### Study population

All PAT data were collected from recruits between six months and eight weeks before they began police training. In order to be eligible to begin the PAT, all trainees were required to meet RNZPC entry requirements for age, moral/ethical character, and health clearance from a General Practitioner. The criteria for inclusion in the study analysis were a) eligibility to attend the Police College, including obtaining a passing PAT score, and b) initiation of training at the Police College following a successful PAT. There were no exclusion criteria.

### Measurements

Height and weight were collected by NZ Police College nursing staff upon trainee entry. The PAT was performed between six months and eight weeks prior to the start of training and consisted of a 2.4 km run, a maximum vertical jump, maximum repetitions of pushups and maximal grip strength.

#### 2.4 Km run

The run event was performed on a level-surface, comprising a 400 m outdoor track. Pace was self-selected, and recruits were provided with their current time at 200 m intervals throughout the event. Recruits were encouraged to complete the event as fast as possible, with times recorded by NZ Police College staff.

#### Vertical jump

Before the vertical jump was performed, standing height was measured. Trainees were required to stand, with feet flat and hands linked together, and reach up as high on possible on a standardized, graduated, and vertically marked height measurement platform. This height was recorded. The trainee then jumped as high as possible and the highest mark reached with either hand was recorded in cm. The standing height measure was subtracted from the jump height, resulting in the final recorded score. Three attempts were permitted, with the highest being counted for the final score.

#### Pushups

Uniform hand placement for pushups was achieved with the following procedure: candidates gently rested their hands on the ground with the thumbs fully extended while lying prone. The thumbs were brought in-line with tip of the acromion, and the other fingertips pointed forwards. The position could then be shifted laterally, but not forwards or backwards, until the elbow reached approximately 90 degrees of flexion. Once this hand position was located, trainees were not permitted shift their hands. The start position was taken by the trainee locking out their arms from the set position and raising the trunk and legs into a straight incline, with only the toes and hands touching the ground. For a pushup repetition to be counted, the trainee lowered to 90 degrees of elbow flexion and then returned to a position where the elbows were straight. If any part of the body other than the hands or toes contacted the ground, the test was terminated, and the score recorded. Recruits completed as many correct repetitions as possible until a part of the body touched the ground, without time limitation.

#### Grip strength

Grip strength was assessed using a Jamar digital hand dynamometer (Sammons Preston, Boilingbrook IL, USA) with the trainee extending the wrist and elbow, flexing the shoulder to 90 degrees and squeezing as hard as possible. Three attempts were permitted, with the highest score being counted towards the final summed score. The result was recorded in kilograms.

#### The PAT score

The summed score for the PAT is dependent on trainee sex and BMI. A passing score is set at 11 points for both males and females, with trainees also requiring a score of at least 1 point in each event to pass. The PAT scoring accounts for differences in BMI to more fairly account for metabolic capacity; recruits with a higher BMI are awarded additional points if their run time is at the pass threshold or greater. The PAT scoring system is still undergoing evaluation for validation; as such raw data were used for the analyses present within this study, and not PAT scores.

#### Police training

All recruits attend the Royal New Zealand Police College in Porirua, NZ for all 16 weeks of training. The college is located on the South Island, 22 km from Wellington, NZ. Training consisted of police procedure and police studies, defensive tactics, firearms training, vehicle operations training and computer operations training.

### Injury data

Injury data were collected via point-of-care reporting by staff who were unaware of the research when attending to injuries. An injury was defined as an accident or incident in an unplanned and unexpected event with undesirable or unfortunate consequences that harmed a worker in the workplace for the purposes of this study [18]. Any injury of sufficient severity to warrant medical attention from either the RNZPC nurse or physiotherapist was considered and included regardless of how that injury affected the trainee’s ability to continue training. Upon cohort graduation, the research team was provided with the injury data aligned with the trainee’s other measures. Injury was denoted with a “1” score for no injury and with a “0” score for those who sustained an injury. Further details of injuries were limited to body location only, recorded as a “1” for any upper limb injury, a “2” for any lower limb injury and a “3” for any trunk (including the neck) injury. Head injuries, lacerations, burns and other non-musculoskeletal or non-peripheral nervous injuries were excluded.

### Statistical method

Data were provided in an Excel (Microsoft Corporation, Redmond WA, USA) spreadsheet, examined by the authors for accuracy, and then imported into SPSS (IBM, Armonk NY, USA) for descriptive analysis. Based on the results of a Kolmogorov-Smirnov test for normality, Mann-Whitney U tests were performed on the fitness testing components to assess differences in scores between injured and uninjured groups. Fitness test data were also divided into quintile ranks based on scores. The relationships between fitness quintiles and injury status were assessed by Spearman’s Correlation analyses. Statistical significance was set at an Alpha level of 0.05 a priori. Those relationships meeting the alpha threshold were then tested for effect size using Cliff’s delta. In this study, the value of Cliff’s Delta was used to indicate the probability that an uninjured trainee will have better PAT component performance than an injured trainee, with a Cliff’s Delta value of + 1.0 indicating that all (100%) uninjured trainees had higher scores than all injured trainees [19].

## Results

A total of 390 records were provided. Of these records, 147 did not have complete entries, leaving 243 subject records available for analysis. A total of 68 injuries occurred in the retained trainee records. There were no significant differences between injured and uninjured groups with respect to age. Significant differences in performance between injured and uninjured groups existed for the following PAT components: pushups and right-hand (R)) grip strength, for all injuries; vertical jump height for lower limb injuries; and all measures of grip strength for trunk injuries (Tables 1, 2 and 3). There were no significant differences between the injured and uninjured groups with respect to upper limb injuries.

**Table 1:** Mean Values of PAT Component Scores by All-Injuries Status

Test	Injured Group Mean (***n*** = 68)	Uninjured Group Mean (***n*** = 175)	***p-***Value	Cliff’s Delta
2.4 km Run (secs)	610.60 ± 62.84	604.52 ± 51.85	0.373	
Vertical Jump (cm)	53.53 ± 9.28	55.06 ± 8.19	0.087	
Pushups (repetitions)	32.94 ± 8.66	35.67 ± 9.04	0.010*	+ 0.11
Left Grip (kg)	49.94 ± 13.79	51.37 ± 11.75	0.136	
Right Grip (kg)	49.61 ± 12.51	52.12 ± 11.17	0.042*	+ 0.22
Combined Grip (kg)	99.55 ± 23.36	103.5 ± 23.19	0.074	

*Indicates *p* < 0.05, from Mann-Whitney U test of difference between groups

**Table 2:** Mean Values of PAT Component Scores by Lower Limb Injury Status

Test	Injured Group Mean (***n*** = 28)	Uninjured Group Mean (n = 175)	***p-***Value	Cliff’s Delta
2.4 km Run (sec)	606.54 ± 68.96	604.52 ± 51.85	0.926	
Vertical Jump (cm)	51.75 ± 7.54	55.06 ± 8.19	0.032*	+ 0.41
Pushups (repetitions)	32.64 ± 7.29	35.67 ± 9.04	0.072	
Left Grip (Kg)	51.31 ± 14.25	51.37 ± 11.75	0.562	
Right Grip (Kg)	49.52 ± 10.77	52.12 ± 11.17	0.136	
Combined Grip (Kg)	100.84 ± 23.31	103.5 ± 23.19	0.282	

*Indicates *p* < 0.05, from Mann-Whitney U test of difference between groups

**Table 3:** Mean Values of PAT Scores by Trunk Injury Status

Test	Injured Mean (***n*** = 13)	Uninjured Mean (n = 175)	***p-***Value	Cliff’s Delta
2.4 km Run (sec)	611.08 ± 67.67	604.52 ± 51.85	0.571	
Vertical Jump (cm)	52.54 ± 7.87	55.06 ± 8.19	0.432	
Pushups (repetitions)	31.69 ± 8.61	35.67 ± 9.04	0.171	
Left Grip (Kg)	42.08 ± 9.89	51.37 ± 11.75	0.011*	+ 0.80
Right Grip (Kg)	42.86 ± 11.79	52.12 ± 11.17	0.008*	+ 0.83
Combined Grip (Kg)	84.95 ± 21.45	103.5 ± 232.27	0.007*	+ 0.80

*Indicates *p* < 0.05, from Mann-Whitney U test of difference between groups

### All injuries

The results of Mann-Whitney U tests for all injury types, comparing mean PAT component scores for injured and uninjured trainees, are shown in Table 1. Differences between the groups, based on all injuries, in mean pushup test and R) Grip strength scores reached statistical significance. Differences for the vertical jump and combined grip strength trended towards significance but did not reach the Alpha threshold for this categorization of injury.

**Fig. 1:**
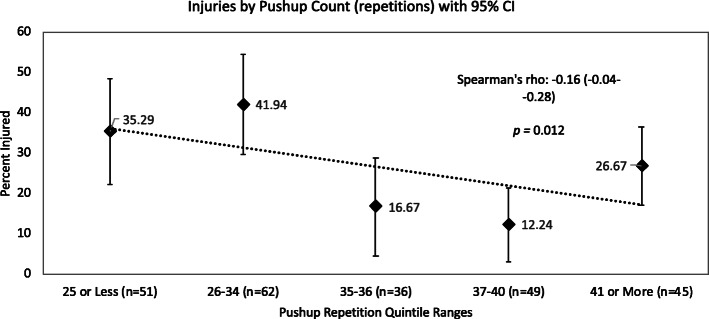
All-Injuries Percentage by Performance Quintile, Pushup Test

**Fig. 2:**
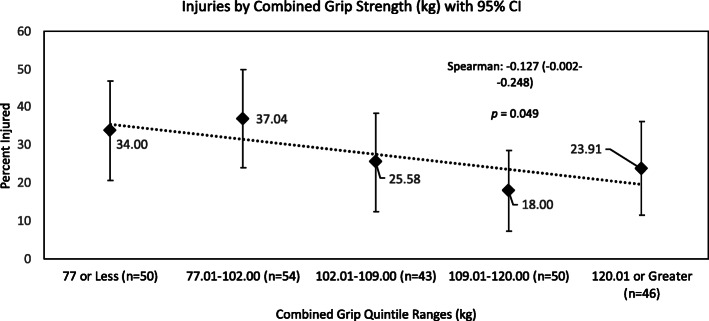
All-Injuries Percentage by Performance Quintile, Combined Grip Strength

**Fig. 3:**
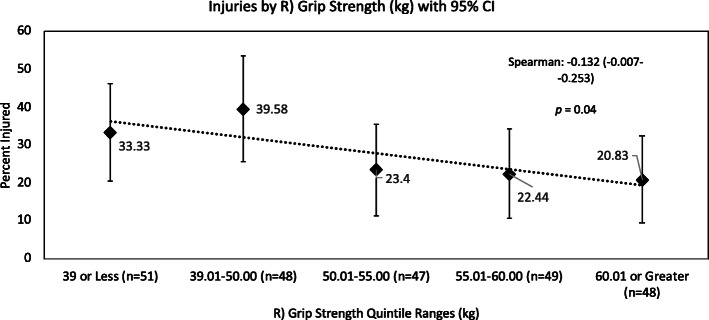
All-Injuries Percentage by Performance Quintile, R) Grip Strength

Relationships between performance quintiles and all-injuries counts are shown in Figs. 1, 2, 3. The relationships between all-injuries risk and pushup test, R) Grip Strength and combined grip strength scores all reached significance in the Spearman’s correlation analyses. None of the other PAT components were significantly related to all-injuries risk. Table 1.

### Upper limb injuries

A total of 26 upper limb injuries were reported in the retained trainee records. None of the PAT component measures were significantly associated specifically with upper limb injury risk.

### Lower limb injuries

A total of 28 lower limb injuries were reported in the retained trainee records. The mean difference in vertical jump test scores between the group sustaining a lower limb injury and the group not sustaining a lower limb injury reached statistical significance. Differences between these groups for the pushup test trended towards significance, but ultimately no other PAT component scores were significantly associated with lower limb injury risk, as seen in Table 2.

### Trunk injuries

A total of 13 trunk injuries were reported in the retained trainee records. The mean differences in all grip strength test scores between the group who sustained a trunk injury when compared to the group that did not reached statistical significance. No other PAT component scores were closely associated with trunk injury risk, as seen in Table 3.

## Discussion

The aim of this study was to investigate relationships between PAT component test performance and risk of injury during police recruit training. Injured and uninjured groups had significant differences in PAT component scores for some, but not all test components. Specifically, those PAT components that were significantly associated with injury risks were the pushup and R) grip strength tests, for all-injuries risk, the vertical jump height for lower limb injury risk only, and all measures of grip strength for trunk injury risk.

Cliff’s delta calculations for the effect size of significant associations between fitness and injury varied significantly, ranging from a modest effect size of 0.11, for the association between pushups and all-injuries risk, to large effect sizes (0.80–0.83) for associations between all measures of grip strength and trunk injury risk [19]. The effect sizes in the grip strength associations with trunk injury risk are especially interesting given that trunk injuries accounted for the smallest number of injuries at 13, reducing the sample size in statistical calculations. A moderate effect size of 0.40 existed for the association between vertical jump and lower limb injury risk. The strength of the associations between these PAT components and injury risks, when compared to the absence of significant associations for other PAT components like run time, could indicate the presence of a ceiling effect. Trainees may be more effectively prepared for running tasks prior to entering training relative to the run time standard in place.

### 2.4 Km run

Timed running events over a fixed distance have been very closely associated with injury risk in military trainee populations [20]. Further evidence suggests that metabolic fitness (as measured by a run) is especially valid in these training environments because military training settings rely on fixed-workload training; less fit trainees must either work at a much higher percentage of their maximal capacity or take longer to complete training events, exposing them to greater risk of injury [21, 22]]. However, if training is self-paced, the strength of the above association can be obscured. Additionally, ability-based training, in which cohorts are divided into smaller teams which all complete tasks at similar, graded levels of intensity, may also obscure the association. Lastly, if trainees are at a high level of fitness generally, or are exceptionally well prepared for one event, such as the run, a ceiling effect may also reduce the effectiveness of the event to predict injury. This may be especially relevant in our study as those who may have been at greatest risk of injury, as identified by their run time, may have been eliminated by the cut-off score for entry to training. As such, our results did not show a significant association between run time and injury risk.

Finally, it should be noted that metabolic fitness and aerobic capacity are important measures for LEOs regardless of musculoskeletal injury risk, given the risk of cardiovascular disease in the police population and the association between cardiovascular fitness and reduced risk of disease [23].

### Vertical jump

Vertical jump test scores were correlated with lower limb injury risk. Previous research specific to the police population has also found the vertical jump test to be associated with risk of injury [6]. This association may be reflective of police occupational tasks; short bouts of high-intensity activity requiring maximal exertion, such as those actions performed during usage of defensive tactics.

### Pushups

Pushup scores were correlated with all-injuries risk but were not associated with risk of injury in a specific body location. Previous research in military cohorts has also found an association between pushup performance and risk of injuries of any kind or of the lower limb [13, 24], not just of the upper limb, as may seem intuitive. This phenomenon could be due to the limitations of our sample but may also suggest that pushup tests are reflective of more global muscular capability. As has been noted in previous research, muscular fitness is crucial in tactical occupational performance [14, 25]; a more fit trainee or operator has a greater fitness reserve, allowing for tolerance of a greater volume of physical tasks with less injury risk because their fatigue threshold is higher than that of less fit trainees or operators [20]. While especially evident in military training that deliberately places trainees under extreme levels of fatigue [26], the same factors may be at play in police training, especially if a block of training features multiple physically taxing evolutions with limited rest between bouts.

### Grip strength

Grip strength was analyzed in terms of both component and combined measures. Only R) handed grip strength was significantly associated with all-injuries risk. All other measures of grip strength were predictive only of trunk injuries specifically, including injuries affecting the neck. These results reinforce findings first reported in this population by Orr, et al. in a cohort of Australian LEO [16]. Hand dominance likely plays a significant role in this association and may explain why R) grip strength was associated with all-injuries risk while other measures of grip strength were predictive only of trunk injury risk. Grip strength may be a correlate of police-specific task performance, such as negotiation of an obstacle course, in which weak grip may increase risk of falls or hard landings, or in defensive tactics training, in which weak grip may impair the trainee’s ability to subdue their opponent, exposing them to additional forces from the assailant. The dependence on grip to complete these tasks may explain the large effect sizes seen in the between-group analyses for trunk injuries (Table 3). As mentioned above, excessive training, either voluntarily or for disciplinary purposes, may lead to increased injury risk through fatigue. Highly fit trainees may engage in more additional voluntary training or may be more willing to take physical risks in training, potentially explaining the U-shaped curves.

### Performance percentile analyses: all injuries risk

Although the associations between scores from some PAT components (pushups, combined grip strength, R] grip strength) and all injuries risk reached statistical significance (Figs. 1, 2 and 3), all performance ranking attempts for PAT components, to form percentiles of performance, revealed that a large number of trainees all scored very closely to one another. This will most likely have obscured and weakened any associations between all-injuries risk and percentiles of performance.

### Limitations

This study is not without its limitations; missing dataset entries reduced the number of trainee records eligible for inclusion. Also, the time between PAT administration and entry into police college varied between trainees, meaning that fitness of the trainee on entry into police college may not be what was reflected on their PAT. Further information as to what phase of training a candidate was in when their injury occurred could strengthen associations between fitness test performance and injury by refuting or verifying hypothesized underlying mechanisms. Further research in this field could also determine if pass/fail thresholds in place are adequately targeting injury thresholds if desired and investigating the relationship between fitness and severity of injury sustained. Investigating the effect of individual motivation on both the fitness assessment and desire to complete Police College could also determine how significant this individual-specific confounding variable may impact the relationships between fitness and injury.

## Conclusions

Our results suggest a modest, but significant relationship between some measures of the PAT and injury risk during police recruit training. They also suggest that one PAT component, namely the 2.4 km run, which has previously been reported as a predictor of injury risk, may not play a role in predicting injury risk within this population. However, it is possible this is because most recruits demonstrated they could perform at an acceptable level on this test prior to entry to training. These results agree with literature investigating similar strength and muscular endurance measures (grip, vertical jump, pushups) in police recruit populations, suggesting that these measures may be directly relevant to training success free of injury or indicate an underlying mechanism governing fitness and injury risk in LEO. Further research aimed at uncovering causal links and drawing from more robust data is necessary to confirm our findings.

## Data Availability

The dataset(s) supporting the conclusions of this article are not publicly available as data were obtained from a law enforcement agency, and as per the research ethics provisions, individual participant (the fitness assessment and injury reports) data cannot be released without a specific request to, and approval from, the sponsoring agency. To make a request, or for further information, please contact Dr. Rob Orr, Bond University Tactical Research Unit; rorr@bond.edu.au.

## Abbreviations

LEO: Law Enforcement Officer
LEA: Law Enforcement Agency
AUD: Australian Dollar
NZ: New Zealand
BMI: Body Mass Index
PAT: Physical Ability Test
BUHREC: Bond University Human Research Ethics Committee
USA: United States of America
